# Liver DNA methylation of *FADS2* associates with *FADS2* genotypex

**DOI:** 10.1186/s13148-019-0609-1

**Published:** 2019-01-17

**Authors:** Paula Walle, Ville Männistö, Vanessa Derenji de Mello, Maija Vaittinen, Alexander Perfilyev, Kati Hanhineva, Charlotte Ling, Jussi Pihlajamäki

**Affiliations:** 1 Department of Clinical Nutrition, Faculty of Health Sciences, Institute of Public Health and Clinical Nutrition, University of Eastern Finland, 70210 Kuopio, Finland; 2 Department of Medicine, University of Eastern Finland and Kuopio University Hospital, 70211 Kuopio, Finland; 3 Epigenetics and Diabetes Unit, Department of Clinical Sciences, Lund University Diabetes Centre, 205 02 Malmö, Sweden; 4 Clinical Nutrition and Obesity Center, Kuopio University Hospital, 70211 Kuopio, Finland

**Keywords:** Non-alcoholic fatty liver disease, FADS2, DNA methylation, Epigenetics, mRNA expression, Liver, Delta-6 desaturase

## Abstract

**Background:**

Non-alcoholic fatty liver disease has been associated with increased mRNA expression of *FADS2* in the liver and estimated activity of delta-6 desaturase in serum, encoded by the *FADS2* gene. Since DNA methylation in the *FADS*1/2/3 gene cluster has been previously linked with genetic variants and desaturase activities, we now aimed to discover factors regulating DNA methylation of the CpG sites annotated to *FADS1*/*2* genes.

**Methods:**

DNA methylation levels in the CpG sites annotated to *FADS2* and *FADS1* were analyzed from liver samples of 95 obese participants of the Kuopio Obesity Surgery Study (34 men and 61 women, age 49.5 ± 7.7 years, BMI 43.0 ± 5.7 kg/m^2^) using the Infinium HumanMethylation450 BeadChip (Illumina). Associations between DNA methylation levels and estimated delta-6 and delta-5 desaturase enzyme activities, liver histology, hepatic mRNA expression, *FADS1*/*2* genotypes, and erythrocyte folate levels were analyzed.

**Results:**

We found a negative correlation between DNA methylation levels of cg06781209 and cg07999042 and hepatic *FADS2* mRNA expression (both *p* < 0.05), and with estimated delta-6 desaturase activity based on both liver and serum fatty acids (all *p* < 0.05). Interestingly, the methylation level of cg07999042 (*p* = 0.001) but not of cg06781209 (*p* = 0.874) was associated with *FADS2* variant rs174616.

**Conclusions:**

Genetic variants of *FADS2* may contribute to the pathogenesis of non-alcoholic fatty liver disease by modifying DNA methylation.

**Electronic supplementary material:**

The online version of this article (10.1186/s13148-019-0609-1) contains supplementary material, which is available to authorized users.

## Background

Non-alcoholic fatty liver disease (NAFLD) refers to a wide spectrum of liver damage that ranges from simple steatosis to liver cirrhosis, affecting approximately 24% of global population [1]. Non-alcoholic steatohepatitis (NASH) is characterized by hepatocyte damage and inflammation in addition to steatosis. In up to 40% of NASH patients, the disease progresses to liver fibrosis, and the patients have an increased risk of end-stage liver disease and liver-related mortality [1–3]. Due to high prevalence of NAFLD and its association with insulin resistance, type II diabetes, metabolic syndrome, dyslipidemia, and cardiovascular diseases, it is also a major contributor to the cardiovascular risk in the population [1–4].

The risk of NAFLD and NASH is affected by genetic and lifestyle factors that associate with lipid metabolism [5–8]. In fact, multiple alterations in fatty acid composition of the liver are linked with NAFLD and NASH, and changes in the transcription of genes regulating fatty acid metabolism may play a role in the pathogenesis of NAFLD [7, 9–14]. Recently, the progression of NAFLD has also been linked with changes in epigenetic mechanisms, including DNA methylation [15–23]. Because changes in DNA methylation affect gene expression and are known to be regulated by both genetic variants and nutritional factors, such as folate [17, 24–30], DNA methylation is a potential step in gene-diet interaction.

We have previously shown that NASH associates with increased mRNA expression of fatty acid desaturase (*FADS*)2 gene in the liver and the activity of delta-6 desaturase, encoded by *FADS2* [12]. Moreover, Howard et al. suggested that gene variants in the *FADS1/2*/*3* gene cluster affect desaturase activities through altered DNA methylation [28]. Thus, we investigated the associations between liver DNA methylation levels in the *FADS2* and *FADS1* loci with delta-6 desaturase and delta-5 desaturase activities, *FADS1* and *FADS2* mRNA expression, *FADS1*/*2* genotypes, and the levels of erythrocyte folate.

## Methods

### Subjects in the Kuopio Obesity Surgery Study

All patients undergoing obesity surgery in Kuopio University Hospital are recruited into an ongoing study investigating metabolic consequences of obesity surgery [31]. The present analysis includes baseline data from 95 people (34 men and 61 women, age 49.5 ± 7.7 years, Table 1), who were accepted for obesity surgery. Criteria for surgery were (1) body mass index (BMI) greater than 40 kg/m^2^, or greater than 35 kg/m^2^ with a significant comorbidity (e.g., type II diabetes); (2) failure of dietary and drug treatments to reduce weight; and (3) no other contraindication for the operation. Blood samples were drawn after an overnight of fasting. People with alcohol consumption of > 2 doses per day or more or people with previously diagnosed liver diseases not related to obesity were excluded from the study. As part of the surgery protocol, subjects were instructed to follow a preoperative very low-calorie diet for an average of 4 weeks. Patients consumed special products designed for a very low-calorie diet, and the daily energy intake was aimed to be 600–800 kcal. The study protocol was approved by the Ethics Committee of the Northern Savo Hospital District, and it was performed in accordance with the Helsinki Declaration. Written informed consent was obtained from all the participants.

**Table 1:** Clinical characteristics of the study subjects (*n* = 95)

	Mean ± SD
Sex (m/f)	34/61
Age (y)	49.5 ± 7.7
BMI (kg/m^2^)	43.0 ± 5.7
ALT (U/l)	45.6 ± 34.3
Fasting glucose (mmol/l)	6.5 ± 2.1
Fasting insulin (mU/l)	18.7 ± 13.5
Total cholesterol (mmol/l)	4.2 ± 0.9
HDL cholesterol (mmol/l)	1.0 ± 0.2
LDL cholesterol (mmol/l)	2.4 ± 0.8
Triglycerides (mmol/l)	1.6 ± 0.7

Data presented as mean ± SD

### Clinical measurements and laboratory determinations

BMI was calculated as weight (in kilograms) divided by height (in meters) squared. The serum glucose concentration was measured by enzymatic hexokinase photometric assay (Konelab Systems Reagents; Thermo Fischer Scientific, Vantaa, Finland). Serum insulin concentration was determined by immunoassay (ADVIA Centaur Insulin IRI, no. 02230141; Siemens Medical Solutions Diagnostics, Tarrytown, NY). Cholesterol, high-density lipoprotein (HDL) cholesterol, and triglyceride concentrations from the whole serum were assayed by standard automated enzymatic methods (Roche Diagnostics, Mannheim, Germany). Plasma alanine aminotransferase (ALT) concentration was determined using kinetic International Federation of Clinical Chemistry methods (Roche Diagnostics, Mannheim, Germany). Erythrocyte folate levels were measured in the fasted state using an electrochemiluminescence immunoassay (Roche Diagnostics).

### Genome-wide DNA methylation analysis in human liver

DNA methylation was assessed according to previously described methods [32] using the Illumina Infinium HumanMethylation450 BeadChip, from which CpG sites annotated to *FADS1* and *FADS2* genes, including 18 sites annotated to *FADS2* and 29 sites annotated to *FADS1,* were measured. The genome-wide DNA methylation data from this study population has previously been published [22, 32]. As described earlier, the raw methylation data in β values were converted to *M* values {*M* = log2[β/(1-β)]}, which were then used for statistical tests, and to easier interpret the results, *M* values were reconverted to β values which were used for tables and creating figures [32].

### Assessment of serum and adipose tissue fatty acid composition and estimation of enzyme activities

In Kuopio Obesity Surgery Study (KOBS), the serum samples were extracted with chloroform–methanol (2:1) and the different lipid fractions, triglycerides (TG), cholesteryl esters (CE), and phospholipids (PL) were separated by solid-phase extraction with an aminopropyl column. Fatty acids were analyzed according to previously described methods [33, 34]. We also measured liver fatty acid composition in CE and TG using the same gas chromatograph method in samples (*n* = 19) that were previously extracted for liver NMR analysis [35].

The results of FA analysis are expressed as molar percentages (mol/mol of all fatty acids). The enzyme activities in serum CE, TG, and PL were estimated as product-to-precursor ratios of individual FAs in all lipid fractions. Delta-5-desaturase was estimated as the ratio of 20:4 n-6/20:3 n-6. Delta-6-desaturase, the ratio of 18:3 n-6/18:2 n-6, was calculated only in CE and TG due to a naturally low proportion of 18:3 n-6 in PL.

### Genotype analyses

The variants rs174547 (*FADS1*) and rs174616 (*FADS2*) were genotyped from DNA samples of the KOBS study using the TaqMan SNP Genotyping Assay (Applied Biosystems, Foster City, CA, USA) according to their protocol. These SNPs were chosen for the current analysis because of their previous association with PUFA levels and desaturase activities [36–39].

### TruSeq targeted RNA expression

Custom gene panel (Illumina, San Diego, CA, USA) was used for measuring the mRNA expression levels of *FADS1* and *FADS2* in human liver according to instructions provided by the manufacturer using MiSeq system (Illumina, San Diego, CA, USA), as described previously [40]. The expression levels for each gene per sample in the custom gene panel were normalized based on the total number of aligned reads of the corresponding sample and the results are shown as percentage of total transcript reads.

### Liver histology

Liver biopsies in the KOBS study were obtained during the obesity surgery using a Trucut needle (Radiplast AB, Uppsala, Sweden) or as a wedge biopsy. Overall histological assessment of liver biopsy samples was performed by one pathologist according to the standard criteria [41, 42]. Histological diagnosis was classified into three categories: (1) normal liver without any steatosis, inflammation, ballooning or fibrosis; (2) simple steatosis (steatosis > 5%) without evidence of hepatocellular ballooning, inflammation or fibrosis; and (3) NASH. Chronic hepatitis B and C were excluded using serology if ALT levels were elevated prior to the surgery. Hemochromatosis was excluded by histological analysis of liver biopsies, and by normal serum ferritin levels in subjects that had elevated serum ALT level.

### LC–MS analysis

Plasma methionine and glycine betaine levels were determined by UHPLC-qTOF-MS system (Agilent Technologies, Waldbronn, Karlsruhe, Germany), using hydrophilic interaction chromatography and positive (+) electrospray ionization. The detailed analytical parameters have been reported earlier [43]. The data acquisition software was MassHunter Acquisition B.04.00 (Agilent Technologies), and the data were evaluated with MassHunter Qualitative Analysis B.05.00 (Agilent Technologies, USA). Methionine and glycine betaine were identified based on authentic chemical standards.

### Statistical analysis

Data are presented as mean ± SD, unless otherwise stated. The final number of subjects in each statistical model and figure ranged between 95 and 19 depending on availability of data. To assess differences in clinical characteristics and DNA methylation between the study groups, one-way ANOVA or a one-way Welch ANOVA with Bonferroni post-hoc test was used. A logarithmic or inverse transformation was performed for skewed variables after assessing normality graphically. Spearman correlation coefficient was used for correlation analyses, and the statistical significance was corrected for multiple comparisons using the Benjamini–Hochberg procedure with a false discovery rate (FDR) of 0.25. The IBM SPSS Statistics for Windows software, Version 24 (IBM Corp., Armonk, NY, USA), was used for statistical analyses.

## Results

### *FADS2* DNA methylation associates with estimated desaturase enzyme activities

**Fig. 1:**
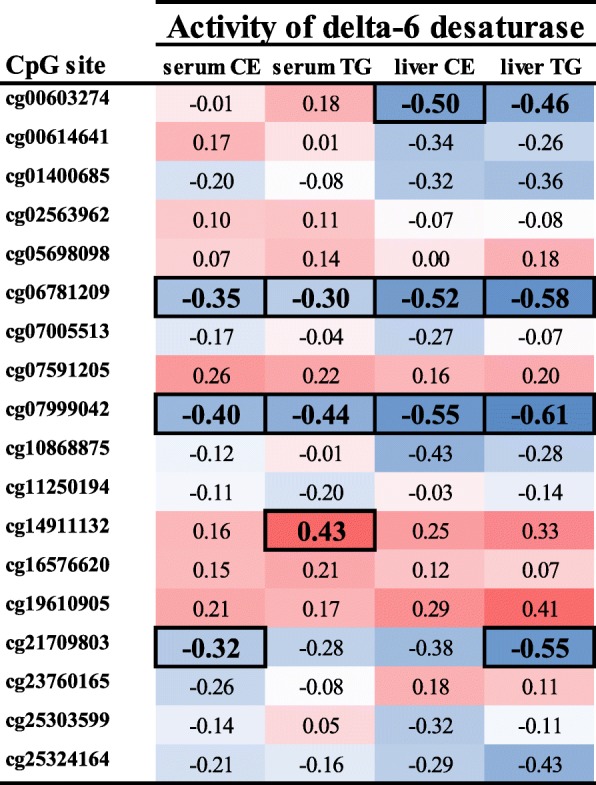
Association between estimated delta-6 desaturase activity in serum and liver and DNA methylation of CpG sites in *FADS2*. A Spearman’s correlation was run to assess the relationship between methylation levels and delta-6 desaturase activity (*n* = 49 for serum and *n* = 19 for liver). Data is presented as Spearman correlation coefficient, and associations with a nominal *p* value < 0.05, which remained significant after correction for multiple testing using the Benjamini-Hochberg procedure with FDR 0.25, are indicated by boxes around the correlation coefficient. Positive correlations indicated with red and negative correlations with blue. Activity of delta-6 desaturase was estimated by a ratio of 18:3 n-6/18:2 n-6 in cholesteryl esters (CE) and triglycerides (TG)

The characteristics of the study cohort are presented in Table 1. First, we selected the CpG sites annotated to *FADS1* and *FADS2* gene regions from the genome-wide DNA methylation data published before [22, 32]. Using this information, we investigated in which of these CpG sites DNA methylation correlated with estimated delta-6 desaturase and delta-5 desaturase enzyme activities based on liver and serum fatty acids analysis (data for individual CpG sites shown in Fig. 1 for *FADS2* and Additional file 1 for *FADS1*). As a result, we discovered that methylation at two CpG sites in the *FADS2*, cg06781209 and cg07999042, correlated significantly with estimated activity of delta-6 desaturase in both liver and serum in all lipid fractions (all *p* < 0.05); therefore, they were selected for further analysis. As for DNA methylation in *FADS1*, some significant correlations were found (Additional file 1), but because no CpG site correlated with delta-5 desaturase activity in all lipid fractions, we focused analyses on *FADS2* methylation.

### *FADS2* DNA methylation associates with *FADS2* mRNA expression in the liver

**Fig. 2:**
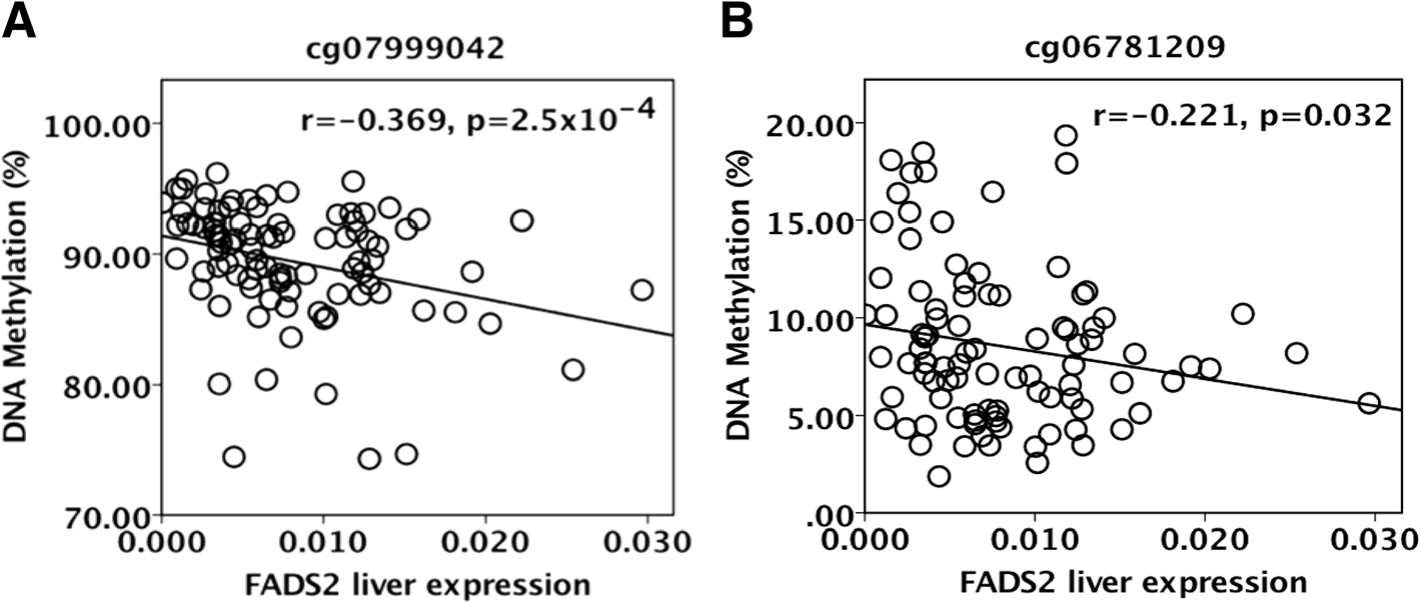
*FADS2* DNA methylation associates with hepatic mRNA expression of *FADS2*. Scatterplots demonstrating correlations between methylation levels of cg06781209 (**a**) and cg07999042 (**b**) and the mRNA expression of *FADS2* in liver. Spearman correlation coefficient was used for correlation analyses, and the statistical significance was corrected for multiple comparisons using the Benjamini–Hochberg procedure with a false discovery rate (FDR) of 0.25. Nominal *p* values are presented

For a DNA methylation at a certain CpG site to regulate transcription, the assumption is that methylation and transcription correlate negatively. In fact, *FADS2* mRNA expression in the liver had a negative correlation with the methylation level of both cg07999042 (*r* = − 0.369, *p* = 2.5 × 10^−4^, Fig. 2) and cg06781209 (*r* = − 0.221, *p* = 0.032) suggesting that these CpG sites could be regulatory.

### Genotype is associated with *FADS2* DNA methylation levels at some CpG sites annotated to *FADS2*

**Fig. 3:**
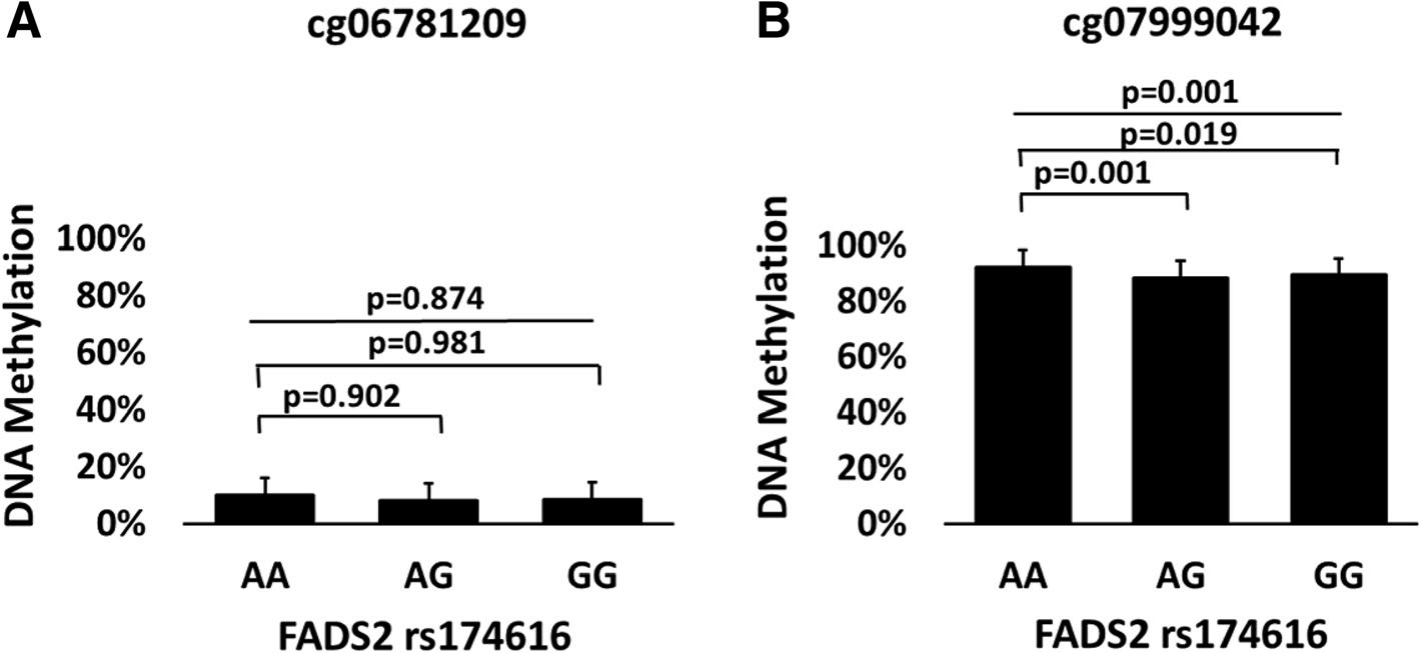
*FADS2* DNA methylation levels according to groups based on *FADS2* genotype. Panels (**a**) and (**b**) show DNA methylation levels in cg06781209 and cg07999042 according to *FADS2* genotype (*n* = 88). Statistical significance was calculated with one-way ANOVA or a one-way Welch ANOVA with Bonferroni post-hoc test and nominal *p* values are presented

We have previously linked genetic variation of *FADS2* and *FADS1* to estimated delta-6 and delta-5 desaturase activities in adipose tissue [40], and Howard et al. suggested that the association between genetic variation in the *FADS* gene cluster and altered fatty acid metabolism is a result of altered DNA methylation [28]. Thus, we investigated if SNPs in *FADS2* and *FADS1* associated with methylation levels of CpG sites in this gene cluster. Interestingly, we discovered that the *FADS2* variant rs174616 had a significant association with the methylation level of cg07999042 (*p* = 0.001 in ANOVA), but not with the methylation level of cg06781209 (*p* = 0.874 in Welch ANOVA, Fig. 3a, b, Additional file 2). Bonferroni post-hoc test revealed that subjects who had the major allele G of the *FADS2* polymorphism had significantly lower methylation levels of cg07999042 than those with the AA genotype (88.3 ± 4.97% vs. 92.3 ± 2.40%, *p* = 0.001 for AG vs. AA, respectively, and 89.4 ± 4.05% vs. 92.3 ± 2.40%, *p* = 0.019 for GG vs. AA, Additional file 2). There were no significant differences in clinical and metabolic parameters between subjects with different *FADS2* genotypes (Additional file 3). *FADS1* variant rs174547 associated with methylation levels at some CpG sites annotated to the *FADS1* gene (Additional file 4). Subjects with the TT genotype had higher HDL cholesterol levels compared to those with CC genotype (*p* = 0.041), but otherwise there were no significant differences between subjects with different *FADS1* genotypes. However, we concentrated on the *FADS2* methylation because of its association with mRNA expression and estimated enzyme activities (see above).

### Association between DNA methylation of *FADS2* and liver steatosis

Since we have previously shown in this study population that NASH associates with altered delta-6 desaturase activity [12], we wanted to investigate if the histological characteristics of NAFLD correlate also with *FADS1*/*2* DNA methylation levels in liver. Spearman correlation analysis showed that both cg06781209 and cg07999042 associated negatively with the grade of steatosis in liver (*n* = 72, nominal *p* < 0.05 for both, Additional file 5), but the correlation did not reach statistical significance after correcting for multiple testing (FDR 0.25). Other histological features of NASH, such as lobular inflammation or hepatocellular ballooning, did not show association with the methylation levels of cg06781209 or cg07999042. The Spearman correlation coefficients for all CpG sites annotated to *FADS1*/*2* methylation and liver histology are presented in Additional file 6.

### Association between DNA methylation levels and erythrocyte folate level

Since our earlier results suggests that hypomethylation in the liver of diabetic subjects could be explained by reduced folate levels in this study cohort [32], we investigated if the erythrocyte folate levels associate with DNA methylation levels in the *FADS2*. We observed a nominally significant correlation between methylation levels of cg06781209 and levels of erythrocyte folate (nominal *p* value of 0.025, Spearman correlation, Additional file 5), but the association did not remain significant after correcting for multiple testing. Further, we divided the subjects into three groups according to erythrocyte folate levels (1st tertile 1076.0; 613.0–1279.0, (median; min-max), 2nd tertile 1417.0; 1280.0–1573.0, 3rd tertile 1939.0;1593.0–2986.0). The groups did not differ in clinical characteristics (Additional file 7). In this preliminary analysis, we observed that there was a borderline significant association between methylation levels of cg06781209 and erythrocyte folate levels (nominal *p* value of 0.050 in ANOVA, Additional file 8). Other nutrients potentially regulating the methylation pathway, such as plasma glycine betaine or l-methionine, did not significantly correlate with DNA methylation levels in the *FADS2* (Additional file 5).

## Discussion

In this study, we aimed to identify factors behind altered fatty acid desaturase activities in NAFLD, following the findings that estimated desaturase enzyme activities in serum and *FADS2* mRNA expression in the liver are higher in individuals with NASH [12]. We found an association of higher hepatic *FADS2* mRNA expression and serum delta-6 desaturase activity with lower levels of DNA methylation in CpG sites annotated to *FADS2*. Moreover, DNA methylation levels of *FADS2* were linked with the *FADS2* genotype, suggesting a novel mechanism for the genetic contribution of *FADS2* genotypes in the pathogenesis of NAFLD.

An important finding of this study was that high estimated delta-6 desaturase activity, observed in subjects with NASH [12], associated with low DNA methylation of two CpG sites annotated to *FADS2*, cg06781209 and cg07999042. The first CpG site, cg06781209, is located in a CpG-rich region (CpG island) within the first 1500 bp upstream from the transcription start site, in a potential gene enhancer region indicated by monomethylation of histone H3 lysine4 (H3K4me1) and acetylation of H3K27 (H3K27Ac) (the ENCODE tracks, Additional file 9) [44, 45]. Moreover, cg06781209 is in an area with several transcription factor binding sites: POLR2A, YY1, CHD2, RELA, CHD1, TAF1, CTCF, SIN3A, and TCF7L2. High peaks of H3K27Ac indicate activation of transcription in this area. The second significant CpG site, cg07999042, is located in the body of *FADS2,* and it is in the same area as transcription factor binding site for POLR2A. The cg07999042 has previously been associated with the risk of type II diabetes in men with low birth weight [29]. Moreover, altered DNA methylation of several genes has been associated with obesity, type II diabetes, and NASH [15–17, 32, 46, 47], but the role of *FADS2* DNA methylation in NAFLD is unknown.

In this study, we observed that lower DNA methylation levels of both cg06781209 and cg07999042 correlated with not only higher delta-6 desaturase activity in serum but also with higher *FADS2* mRNA expression in the liver. These associations between *FADS2* DNA methylation, mRNA expression, and desaturase activity suggest that alterations in the methylation level of these CpG sites annotated to *FADS2* may affect delta-6 desaturase activities through transcriptional changes, especially considering their location in the area with several transcription binding sites (Additional file 9). Similar findings have been previously demonstrated for other genes involved in fatty acid metabolism in subjects with NAFLD [18]. However, since we were able to study only associations between DNA methylation, mRNA expression and desaturase activities, the mechanisms need to be further examined in future studies.

We discovered that altered *FADS2* DNA methylation was associated with the *FADS2* variant rs174616. This variant is located in intron 7 of the *FADS2* gene (Additional file 9) and has been associated with alterations in fatty acid metabolism, inflammation, and type II diabetes [39, 40, 48]. In this study, subjects with the major allele G had lower DNA methylation levels of cg07999042 in *FADS2* compared to homozygotes for the minor allele A. Accordingly, we have shown that the subjects with the major allele G have also higher delta-6 desaturase activity, similarly to patients with NASH [12, 40]. Thus, it is plausible that *FADS2* genotype may regulate delta-6 desaturase activity at least partly through DNA methylation. Similar results have been published by Rahbar et al., who reported associations between genotype and DNA methylation in the putative enhancer region within the *FADS* gene cluster [49], and by Howard et al., who suggested that the association between genetic variation in the *FADS* gene cluster and altered fatty acid metabolism is a result of altered DNA methylation [28].

Hepatic steatosis is associated with disturbances in the methylation pathway [23, 50], and altered DNA methylation has been linked with different stages of NAFLD, possibly promoting the disease progression [15–22]. Thus, we wanted to study the associations between DNA methylation and histological features of NAFLD. After correcting for multiple testing, only one CpG site annotated to *FADS1* showed significant correlation to steatosis grade and fibrosis stage (cg16213375). No significant correlations were found for *FADS2*, but both cg06781209 and cg07999042 had a nominally significant correlation with steatosis grade. Based on these results, it would be of interest to study the possible role of DNA methylation of genes regulating fatty acid metabolism in the future. Although altered DNA methylation in several other genes has been associated with NASH and liver fibrosis [15, 16, 18, 20–22], DNA methylation in neither cg06781209 nor cg07999042 in *FADS2* correlated with fibrosis or inflammation in the liver. This supports our suggestion that altered DNA methylation in *FADS2* contributes primarily to altered desaturase activity and possibly liver steatosis in NAFLD, and not to liver inflammation or fibrosis.

We wanted to investigate if DNA methylation levels in *FADS2* associate with erythrocyte folate levels. Folate is a coenzyme in methylation pathway in the liver, affecting the synthesis of the universal methyl donor in DNA methylation, S-adenosyl-methionine [19, 25]. Since folate modulates the availability of methyl donors in DNA methylation, it can affect gene expression and thus influence cell function. Moreover, folate deficiency and changes in S-adenosyl-methionine have been related to hepatic accumulation of triglycerides and alterations in expression of genes involved in fatty acid metabolism [50]. Although reduced folate levels are often associated with low DNA methylation, as we have also published for genes related to type II diabetes [32], in this preliminary analysis we observed only a nominally significant negative correlation between folate levels and DNA methylation of cg06781209. Similar findings have been observed in mice, as Tryndyak et al. reported hypermethylation in response to folate-deficient diet in mice with severe NASH-like liver injury [20]. The discrepancy in these findings and previous studies warrants that the association between erythrocyte folate levels and DNA methylation in liver needs to be investigated in experimental models and in larger study populations. Finally, other nutrients in the methylation pathway, and potentially the amount and quality of fat in the diet [50], may regulate DNA methylation in the liver. For example, amino acids, such as methionine and betaine, are involved in the synthesis of S-adenosyl-methionine and serve as methyl donors [19, 25, 26]. Despite their important role in the methylation pathway and association with NAFLD [51], we found no significant correlations between serum methionine, glycine betaine, and the methylation levels of cg06781209 and cg07999042.

We recognize the following limitations in our study. The sample size was modest; thus, we may not have recognized all relevant associations. As the study was cross-sectional, we cannot conclude that there is a causal relationship between DNA methylation, *FADS2* mRNA expression, and delta-6 desaturase activities. Actual enzyme activities could not be measured with human liver samples. Instead, we used estimated enzyme activities calculated from product-to-precursor ratios as surrogate measures, similarly to previous studies [28, 52, 53]. We acknowledge that because this study was performed in obese subjects, the results might not reflect the DNA methylation in normal-weighed individuals with NAFLD. Moreover, due to strong linkage disequilibrium in the *FADS1*/*2* locus, we acknowledge that the *FADS2* variant rs174616 most likely is a marker for other uncharacterized functional variants.

## Conclusions

In conclusion, we observed a novel association of *FADS2* DNA methylation at selected CpG sites with the *FADS2* variant rs174616. Moreover, DNA methylation levels of these CpG sites correlated with *FADS2* mRNA expression and estimated delta-6 desaturase activity. Thus, we suggest that genetic variants of *FADS2* may contribute to the pathogenesis of NAFLD by modifying fatty acid metabolism through DNA methylation.

## Additional files

Additional file 1: Association between estimated delta-5 desaturase activity in serum and liver and DNA methylation of CpG sites in *FADS1*. (DOCX 26 kb)

Additional file 2: DNA methylation levels in CpG-sites annotated to *FADS2* in groups based on *FADS2* variant rs174616. (DOCX 27 kb)

Additional file 3: Characteristics of the groups based on FADS2 genotype. (DOCX 24 kb)

Additional file 4: DNA methylation levels in CpG-sites annotated to *FADS1* in groups based on *FADS1* genotype. (DOCX 30 kb)

Additional file 5: Correlation of DNA methylation levels of cg06781209 and cg07999042 with clinical parameters and liver histology. (DOCX 25 kb)

Additional file 6: Association between liver histology and DNA methylation of CpG sites annotated to *FADS2* and *FADS1*. (DOCX 29 kb)

Additional file 7: Characteristics of the groups based on erythrocyte folate. (DOCX 27 kb)

Additional file 8: DNA methylation of *FADS2* in groups based on erythrocyte folate. (DOCX 26 kb)

Additional file 9: Association between cg06781209, cg07999042 and rs174616 in the *FADS* gene cluster. (DOCX 135 kb)

## Abbreviations

ALT: Alanine aminotransferase
BMI: Body mass index
CE: Cholesteryl esters
*FADS*: Fatty acid desaturase
HDL: High-density lipoprotein
KOBS: Kuopio Obesity Surgery Study
NAFLD: Non-alcoholic fatty liver disease
NASH: Non-alcoholic steatohepatitis
PL: Phospholipids
TG: Triglycerides
