# Correction to: Liver DNA methylation of *FADS2* associates with *FADS2* genotype

**DOI:** 10.1186/s13148-019-0625-1

**Published:** 2019-03-12

**Authors:** Paula Walle, Ville Männistö, Vanessa Derenji de Mello, Maija Vaittinen, Alexander Perfilyev, Kati Hanhineva, Charlotte Ling, Jussi Pihlajamäki

**Affiliations:** 1 Department of Clinical Nutrition, Faculty of Health Sciences, Institute of Public Health and Clinical Nutrition, University of Eastern Finland, 70210 Kuopio, Finland; 2 Department of Medicine, University of Eastern Finland, Kuopio University Hospital, 70211 Kuopio, Finland; 3 Epigenetics and Diabetes Unit, Department of Clinical Sciences, Lund University Diabetes Centre, 20502 Malmö, Sweden; 4 Clinical Nutrition and Obesity Center, Kuopio University Hospital, 70211 Kuopio, Finland

**Correction to: Clin Epigenetics**

**https://doi.org/10.1186/s13148-019-0609-1**

Following publication of the original article [1], the author reported the title of this article has been misspelled. The correct title is “Liver DNA methylation of *FADS2* associates with *FADS2* genotype”.

It has been corrected in the original article as well.

The publisher apologizes for any inconvenience caused by this error.
