# Supplementary material for: Liver DNA methylation of FADS2 associates with FADS2 genotypex
Source: Clin Epigenetics. 2019 Jan 17;11:10. doi: 10.1186/s13148-019-0609-1 (PMC6337806; doi:10.1186/s13148-019-0609-1)
Supplement: Supplementary file 1 — Association between estimated delta-5 desaturase activity in serum and liver and DNA methylation of CpG sites in FADS1. (DOCX 26 kb) [file 13148_2019_609_MOESM1_ESM.docx]

ADDITIONAL MATERIAL:

**Liver DNA methylation of *FADS2* associates with *FADS2* genotypes.**

Paula Walle^1^, Ville Männistö^2^, Vanessa D. de Mello^1^, Maija Vaittinen^1^, Alexander Perfilyev^3^, Kati Hanhineva^1^, Charlotte Ling^3^, Jussi Pihlajamäki^1,4^

1 Department of Clinical Nutrition, Institute of Public Health and Clinical Nutrition, University of Eastern Finland, Kuopio, Finland.

2 Department of Medicine, University of Eastern Finland and Kuopio University Hospital, Finland

3 Epigenetics and Diabetes Unit, Department of Clinical Sciences, Lund University Diabetes Centre, Malmö, Sweden.

4 Clinical Nutrition and Obesity Center, Kuopio University Hospital, Finland

**Additional file 1.**

|  | **Activity of delta-5 desaturase** | | | | | |
| --- | --- | --- | --- | --- | --- | --- |
| **CpG-site** | **serum CE** | **serum TG** | **serum PL** | **liver CE** | **liver TG** | **liver PL** |
| **cg00786201** | -0.02 | 0.03 | -0.05 | -0.30 | -0.18 | 0.11 |
| **cg02085160** | -0.10 | 0.00 | -0.13 | 0.17 | 0.18 | 0.14 |
| **cg03735013** | -0.07 | -0.01 | -0.07 | 0.19 | 0.39 | 0.33 |
| **cg03921599** | -0.07 | -0.14 | -0.04 | -0.20 | 0.02 | 0.08 |
| **cg05168842** | 0.07 | 0.13 | 0.06 | 0.16 | 0.17 | 0.43 |
| **cg06405978** | 0.12 | -0.04 | 0.13 | 0.26 | 0.13 | 0.10 |
| **cg06985934** | -0.09 | 0.05 | -0.07 | **-0.53** | -0.36 | -0.38 |
| **cg07152460** | -0.04 | 0.08 | -0.07 | 0.02 | -0.12 | 0.02 |
| **cg07689907** | -0.13 | -0.01 | -0.16 | -0.24 | -0.25 | -0.13 |
| **cg07709195** | **-0.46** | **-0.40** | **-0.44** | **-0.54** | **-0.55** | -0.42 |
| **cg09462826** | -0.22 | -0.08 | -0.20 | -0.44 | -0.38 | -0.25 |
| **cg09677638** | 0.18 | 0.22 | 0.20 | 0.33 | 0.11 | 0.18 |
| **cg10515671** | **-0.53** | -0.25 | **-0.49** | -0.42 | **-0.51** | -0.25 |
| **cg11606466** | 0.12 | -0.05 | 0.10 | 0.20 | 0.07 | 0.15 |
| **cg12517394** | 0.03 | 0.07 | 0.03 | 0.09 | 0.00 | 0.14 |
| **cg13100764** | -0.08 | 0.09 | -0.03 | -0.11 | **-0.49** | -0.33 |
| **cg13121120** | -0.22 | -0.01 | -0.19 | -0.43 | -0.28 | -0.14 |
| **cg13475388** | 0.03 | 0.06 | 0.02 | -0.13 | 0.05 | -0.05 |
| **cg14725641** | 0.00 | 0.07 | -0.01 | -0.27 | -0.26 | -0.39 |
| **cg15598662** | -0.07 | 0.12 | -0.03 | -0.02 | -0.03 | -0.07 |
| **cg16213375** | **-0.40** | -0.12 | **-0.34** | **-0.62** | **-0.54** | -0.34 |
| **cg16328381** | -0.06 | -0.13 | -0.10 | 0.13 | -0.11 | -0.31 |
| **cg23992449** | 0.14 | 0.00 | 0.16 | 0.13 | 0.44 | 0.07 |
| **cg24870774** | -0.06 | -0.01 | -0.07 | 0.08 | -0.05 | 0.17 |
| **cg25326896** | **0.32** | 0.15 | 0.27 | 0.32 | 0.35 | 0.36 |
| **cg25401284** | 0.05 | 0.13 | 0.09 | -0.09 | -0.11 | 0.07 |
| **cg25448062** | 0.04 | -0.20 | -0.04 | 0.19 | **0.57** | 0.22 |
| **cg25837350** | 0.11 | -0.02 | 0.10 | 0.03 | 0.16 | 0.04 |
| **cg27173322** | -0.02 | 0.14 | -0.02 | -0.11 | 0.05 | 0.14 |

**Association between estimated delta-5 desaturase activity in serum and liver and DNA methylation of CpG sites in *FADS1*.** A Spearman's correlation was run to assess the relationship between methylation levels and estimated delta-5 desaturase activity (n=49 for serum and n=19 for liver). Data is presented as Spearman correlation coefficient, and associations with a nominal p-value <0.05, which remained significant after correction for multiple testing using the Benjamini-Hochberg procedure with FDR 0.25, are indicated by boxes around the correlation coefficient. Activity of delta-5 desaturase was estimated by a ratio of 20:4 n-6/20:3 n-6 in cholesteryl esters (CE), triglycerides (TG) and phospholipids (PL).
