# Supplementary material for: Liver DNA methylation of FADS2 associates with FADS2 genotypex
Source: Clin Epigenetics. 2019 Jan 17;11:10. doi: 10.1186/s13148-019-0609-1 (PMC6337806; doi:10.1186/s13148-019-0609-1)
Supplement: Supplementary file 2 — DNA methylation levels in CpG-sites annotated to FADS2 in groups based on FADS2 variant rs174616. (DOCX 27 kb) [file 13148_2019_609_MOESM2_ESM.docx]

ADDITIONAL MATERIAL:

**Liver DNA methylation of *FADS2* associates with *FADS2* genotypes.**

Paula Walle^1^, Ville Männistö^2^, Vanessa D. de Mello^1^, Maija Vaittinen^1^, Alexander Perfilyev^3^, Kati Hanhineva^1^, Charlotte Ling^3^, Jussi Pihlajamäki^1,4^

1 Department of Clinical Nutrition, Institute of Public Health and Clinical Nutrition, University of Eastern Finland, Kuopio, Finland.

2 Department of Medicine, University of Eastern Finland and Kuopio University Hospital, Finland

3 Epigenetics and Diabetes Unit, Department of Clinical Sciences, Lund University Diabetes Centre, Malmö, Sweden.

4 Clinical Nutrition and Obesity Center, Kuopio University Hospital, Finland

| **Additional File 2. DNA methylation levels in CpG-sites annotated to *FADS2* in groups based on *FADS2* variant rs174616 (n=88).** | | | | | | | | | | | | | | | | | |  |
| --- | --- | --- | --- | --- | --- | --- | --- | --- | --- | --- | --- | --- | --- | --- | --- | --- | --- | --- |
|  |  |  |  |  | |  |  | |  |  |  | |  | |  | |  |  |
|  | ***FADS2* genotype** | | | | | | | | | | | | | | | | **ANOVA*** |  |
| **CpG site** | **AA (n=20)** | | | **AG (n=41)** | | | | | | **GG (n=27)** | | | | | | | **p-value** |  |
| **cg00603274** | 0.05 | ± | 0.01 | | 0.05 | ± | 0.01 |  | 0.05 | | | ± | 0.01 |  | | 0.077 | | |
| **cg00614641** | 0.05 | ± | 0.01 | | 0.05 | ± | 0.01 |  | 0.05 | | | ± | 0.01 |  | | 0.917 | | |
| **cg01400685** | 0.35 | ± | 0.08 | | 0.33 | ± | 0.06 |  | 0.33 | | | ± | 0.07 |  | | 0.555 | | |
| **cg02563962** | 0.05 | ± | 0.01 | | 0.05 | ± | 0.01 |  | 0.06 | | | ± | 0.01 | *^a^* | | **0.022** | | |
| **cg05698098** | 0.11 | ± | 0.02 | | 0.11 | ± | 0.02 |  | 0.11 | | | ± | 0.01 |  | | 0.886 | | |
| **cg06781209** | 0.10 | ± | 0.06 | | 0.08 | ± | 0.04 |  | 0.08 | | | ± | 0.04 |  | | 0.874 | | |
| **cg07005513** | 0.05 | ± | 0.01 | | 0.05 | ± | 0.01 |  | 0.05 | | | ± | 0.01 |  | | 0.446 | | |
| **cg07591205** | 0.93 | ± | 0.01 | | 0.92 | ± | 0.01 |  | 0.93 | | | ± | 0.01 |  | | 0.138 | | |
| **cg07999042** | 0.92 | ± | 0.02 | | 0.88 | ± | 0.05 | *^a^* | 0.89 | | | ± | 0.04 | *^a^* | | **0.001** | | |
| **cg10868875** | 0.04 | ± | 0.01 | | 0.04 | ± | 0.01 |  | 0.04 | | | ± | 0.01 |  | | 0.406 | | |
| **cg11250194** | 0.84 | ± | 0.04 | | 0.87 | ± | 0.03 | *^a^* | 0.87 | | | ± | 0.03 | *^a^* | | **0.006** | | |
| **cg14911132** | 0.09 | ± | 0.01 | | 0.09 | ± | 0.01 |  | 0.09 | | | ± | 0.01 |  | | 0.532 | | |
| **cg16576620** | 0.03 | ± | 0.00 | | 0.03 | ± | 0.01 |  | 0.03 | | | ± | 0.00 |  | | 0.352 | | |
| **cg19610905** | 0.04 | ± | 0.01 | | 0.06 | ± | 0.01 | *^a^* | 0.06 | | | ± | 0.01 | *^a^* | | **5x10^-6^** | | |
| **cg21709803** | 0.09 | ± | 0.04 | | 0.08 | ± | 0.02 |  | 0.09 | | | ± | 0.03 |  | | 0.428 | | |
| **cg23760165** | 0.03 | ± | 0.01 | | 0.03 | ± | 0.01 |  | 0.03 | | | ± | 0.01 |  | | 0.814 | | |
| **cg25303599** | 0.05 | ± | 0.01 | | 0.05 | ± | 0.01 |  | 0.05 | | | ± | 0.01 |  | | 0.247 | | |
| **cg25324164** | 0.26 | ± | 0.05 | | 0.25 | ± | 0.05 |  | 0.24 | | | ± | 0.04 |  | | 0.644 | | |
| Data presented as mean±SD | | | | | | | | | | | | | | | | |  |  |
| *one-way ANOVA or Welch ANOVA | | | | | | | | | | | | | | | | |  |  |
| *^a^* p<0.05 compared to AA genotype in Bonferroni post hoc analysis | | | | | | | | | | | | | | | | |  |  |
