# Supplementary material for: Liver DNA methylation of FADS2 associates with FADS2 genotypex
Source: Clin Epigenetics. 2019 Jan 17;11:10. doi: 10.1186/s13148-019-0609-1 (PMC6337806; doi:10.1186/s13148-019-0609-1)
Supplement: Supplementary file 3 — Characteristics of the groups based on FADS2 genotype. (DOCX 24 kb) [file 13148_2019_609_MOESM3_ESM.docx]

ADDITIONAL MATERIAL:

**Liver DNA methylation of *FADS2* associates with *FADS2* genotype.**

Paula Walle^1^, Ville Männistö^2^, Vanessa D. de Mello^1^, Maija Vaittinen^1^, Alexander Perfilyev^3^, Kati Hanhineva^1^, Charlotte Ling^3^, Jussi Pihlajamäki^1,4^

1 Department of Clinical Nutrition, Institute of Public Health and Clinical Nutrition, University of Eastern Finland, Kuopio, Finland.

2 Department of Medicine, University of Eastern Finland and Kuopio University Hospital, Finland

3 Epigenetics and Diabetes Unit, Department of Clinical Sciences, Lund University Diabetes Centre, Malmö, Sweden.

4 Clinical Nutrition and Obesity Center, Kuopio University Hospital, Finland

| **Additional File 3. Characteristics of the groups based on *FADS2* genotype.** | | | | | | | | | |  |
| --- | --- | --- | --- | --- | --- | --- | --- | --- | --- | --- |
|  |  |  |  |  |  |  |  |  |  |  |
|  | **FADS2 genotype** | | | | | | | | | |
|  | **AA (n=20)** | | | **AG (n=41)** | | | **GG (n=27)** | | | **p-value** |
|  |  | | |  | | |  | | |  |
| Age (y) | 47.4 | ± | 7.6 | 49.5 | ± | 7.7 | 50.8 | ± | 8.1 | 0.340 |
| BMI (kg/m^2^) | 43.9 | ± | 6.1 | 43.5 | ± | 6.5 | 41.4 | ± | 3.8 | 0.217 |
| ALT (U/l) | 38.7 | ± | 18.0 | 40.9 | ± | 26.3 | 58.6 | ± | 50.4 | 0.130 |
| Fasting glucose (mmol/l) | 6.4 | ± | 1.2 | 6.7 | ± | 2.3 | 6.4 | ± | 2.3 | 0.598 |
| Fasting insulin (U/l) | 19.9 | ± | 11.2 | 19.1 | ± | 16.7 | 18.3 | ± | 10.0 | 0.695 |
| Total cholesterol (mmol/l) | 4.4 | ± | 1.0 | 4.1 | ± | 1.0 | 4.2 | ± | 0.7 | 0.612 |
| HDL cholesterol (mmol/l) | 0.9 | ± | 0.2 | 1.0 | ± | 0.2 | 1.1 | ± | 0.3 | 0.080 |
| LDL cholesterol (mmol/l) | 2.7 | ± | 0.9 | 2.3 | ± | 0.9 | 2.4 | ± | 0.6 | 0.347 |
| Triglycerides (mmol/l) | 1.7 | ± | 0.4 | 1.7 | ± | 0.7 | 1.6 | ± | 0.7 | 0.428 |
| Data presented as mean±SD. |  |  |  |  |  |  |  |  |  |  |
| Statistical significance calculated with ANOVA or Welch ANOVA. | | | | | | | |  |  |  |
