# Supplementary material for: Liver DNA methylation of FADS2 associates with FADS2 genotypex
Source: Clin Epigenetics. 2019 Jan 17;11:10. doi: 10.1186/s13148-019-0609-1 (PMC6337806; doi:10.1186/s13148-019-0609-1)
Supplement: Supplementary file 4 — DNA methylation levels in CpG-sites annotated to FADS1 in groups based on FADS1 genotype. (DOCX 30 kb) [file 13148_2019_609_MOESM4_ESM.docx]

ADDITIONAL MATERIAL:

**Liver DNA methylation of *FADS2* associates with *FADS2* genotype.**

Paula Walle^1^, Ville Männistö^2^, Vanessa D. de Mello^1^, Maija Vaittinen^1^, Alexander Perfilyev^3^, Kati Hanhineva^1^, Charlotte Ling^3^, Jussi Pihlajamäki^1,4^

1 Department of Clinical Nutrition, Institute of Public Health and Clinical Nutrition, University of Eastern Finland, Kuopio, Finland.

2 Department of Medicine, University of Eastern Finland and Kuopio University Hospital, Finland

3 Epigenetics and Diabetes Unit, Department of Clinical Sciences, Lund University Diabetes Centre, Malmö, Sweden.

4 Clinical Nutrition and Obesity Center, Kuopio University Hospital, Finland

| **Additional File 4. DNA methylation levels in CpG-sites annotated to *FADS1* in groups based on *FADS1* genotype (n=88).** | | | | | | | | | | | | | |
| --- | --- | --- | --- | --- | --- | --- | --- | --- | --- | --- | --- | --- | --- |
|  |  |  |  |  |  |  |  |  |  |  |  |  |  |
|  | ***FADS1* genotype** | | | | | | | | | | | | **ANOVA*** |
|  | **CC (n=19)** | | | **CT (n=46)** | | | | **TT (n=23)** | | | |  | **p-value** |
| **cg00786201** | 0.06 | ± | 0.01 | 0.05 | ± | 0.01 |  | 0.05 | ± | 0.01 |  |  | 0.354 |
| **cg02085160** | 0.04 | ± | 0.01 | 0.03 | ± | 0.00 |  | 0.03 | ± | 0.01 |  |  | 0.410 |
| **cg03735013** | 0.04 | ± | 0.01 | 0.04 | ± | 0.00 |  | 0.04 | ± | 0.00 |  |  | 0.068 |
| **cg03921599** | 0.03 | ± | 0.01 | 0.03 | ± | 0.01 |  | 0.03 | ± | 0.01 |  |  | 0.342 |
| **cg05168842** | 0.11 | ± | 0.01 | 0.11 | ± | 0.01 |  | 0.11 | ± | 0.01 |  |  | 0.379 |
| **cg06405978** | 0.04 | ± | 0.01 | 0.04 | ± | 0.01 |  | 0.04 | ± | 0.01 |  |  | 0.936 |
| **cg06985934** | 0.04 | ± | 0.01 | 0.04 | ± | 0.01 |  | 0.03 | ± | 0.01 | *^a^* | *^b^* | **1x10^-4^** |
| **cg07152460** | 0.12 | ± | 0.02 | 0.12 | ± | 0.01 |  | 0.11 | ± | 0.01 |  | *^b^* | **0.035** |
| **cg07689907** | 0.07 | ± | 0.02 | 0.07 | ± | 0.02 |  | 0.06 | ± | 0.01 |  |  | 0.120 |
| **cg07709195** | 0.82 | ± | 0.04 | 0.77 | ± | 0.04 | *^a^* | 0.75 | ± | 0.06 | *^a^* | | **4x10^-5^** |
| **cg09462826** | 0.11 | ± | 0.03 | 0.09 | ± | 0.02 | *^a^* | 0.07 | ± | 0.02 | *^a^* | *^b^* | **2x10^-5^** |
| **cg09677638** | 0.04 | ± | 0.01 | 0.04 | ± | 0.01 | *^a^* | 0.04 | ± | 0.01 |  |  | **0.012** |
| **cg10515671** | 0.68 | ± | 0.10 | 0.62 | ± | 0.07 | *^a^* | 0.53 | ± | 0.07 | *^a^* | *^b^* | **2x10^-6^** |
| **cg11606466** | 0.94 | ± | 0.01 | 0.93 | ± | 0.01 |  | 0.94 | ± | 0.01 |  |  | **0.047** |
| **cg12517394** | 0.04 | ± | 0.01 | 0.05 | ± | 0.01 |  | 0.05 | ± | 0.01 |  |  | 0.284 |
| **cg13100764** | 0.04 | ± | 0.01 | 0.03 | ± | 0.01 |  | 0.04 | ± | 0.01 |  |  | 0.092 |
| **cg13121120** | 0.06 | ± | 0.03 | 0.05 | ± | 0.02 |  | 0.04 | ± | 0.01 | *^a^* | *^b^* | **4x10^-7^** |
| **cg13475388** | 0.03 | ± | 0.00 | 0.03 | ± | 0.01 |  | 0.03 | ± | 0.01 |  |  | 0.806 |
| **cg14725641** | 0.03 | ± | 0.01 | 0.03 | ± | 0.01 |  | 0.03 | ± | 0.01 |  |  | 0.580 |
| **cg15598662** | 0.03 | ± | 0.01 | 0.03 | ± | 0.01 |  | 0.03 | ± | 0.01 |  |  | 0.627 |
| **cg16213375** | 0.21 | ± | 0.07 | 0.18 | ± | 0.05 |  | 0.10 | ± | 0.02 | *^a^* | *^b^* | **5x10^-13^** |
| **cg16328381** | 0.93 | ± | 0.02 | 0.93 | ± | 0.02 |  | 0.93 | ± | 0.01 |  |  | 0.660 |
| **cg23992449** | 0.02 | ± | 0.00 | 0.02 | ± | 0.00 |  | 0.02 | ± | 0.00 |  |  | 0.078 |
| **cg24870774** | 0.03 | ± | 0.01 | 0.03 | ± | 0.01 |  | 0.03 | ± | 0.01 |  |  | 0.447 |
| **cg25326896** | 0.03 | ± | 0.00 | 0.03 | ± | 0.01 |  | 0.03 | ± | 0.00 |  |  | 0.343 |
| **cg25401284** | 0.02 | ± | 0.01 | 0.02 | ± | 0.01 |  | 0.02 | ± | 0.00 |  |  | 0.331 |
| **cg25448062** | 0.95 | ± | 0.01 | 0.95 | ± | 0.02 |  | 0.95 | ± | 0.01 |  |  | 0.101 |
| **cg25837350** | 0.16 | ± | 0.02 | 0.17 | ± | 0.02 |  | 0.17 | ± | 0.03 |  |  | 0.546 |
| **cg27173322** | 0.02 | ± | 0.00 | 0.02 | ± | 0.00 |  | 0.02 | ± | 0.00 |  |  | 0.109 |
| Data presented as mean±SD | | | | | | | | | | | |  |  |
| *one-way ANOVA or Welch ANOVA | | | | | | | | | | | |  |  |
| *^a^* p<0.05 compared to CC genotype in Bonferroni post hoc analysis | | | | | | | | | | | |  |  |
| *^b^* p<0.05 compared to CT genotype in Bonferroni post hoc analysis | | | | | | | | | | | |  |  |
