# Supplementary material for: Liver DNA methylation of FADS2 associates with FADS2 genotypex
Source: Clin Epigenetics. 2019 Jan 17;11:10. doi: 10.1186/s13148-019-0609-1 (PMC6337806; doi:10.1186/s13148-019-0609-1)
Supplement: Supplementary file 5 — Correlation of DNA methylation levels of cg06781209 and cg07999042 with clinical parameters and liver histology. (DOCX 25 kb) [file 13148_2019_609_MOESM5_ESM.docx]

ADDITIONAL MATERIAL:

**Liver DNA methylation of *FADS2* associates with *FADS2* genotype.**

Paula Walle^1^, Ville Männistö^2^, Vanessa D. de Mello^1^, Maija Vaittinen^1^, Alexander Perfilyev^3^, Kati Hanhineva^1^, Charlotte Ling^3^, Jussi Pihlajamäki^1,4^

1 Department of Clinical Nutrition, Institute of Public Health and Clinical Nutrition, University of Eastern Finland, Kuopio, Finland.

2 Department of Medicine, University of Eastern Finland and Kuopio University Hospital, Finland

3 Epigenetics and Diabetes Unit, Department of Clinical Sciences, Lund University Diabetes Centre, Malmö, Sweden.

4 Clinical Nutrition and Obesity Center, Kuopio University Hospital, Finland

| **Additional File 5. Correlation of DNA methylation levels of cg06781209 and cg07999042 with clinical parameters and liver histology.** | | | | | |
| --- | --- | --- | --- | --- | --- |
|  |  |  |  |  |  |
|  | **cg06781209** | |  | **cg07999042** | |
|  | r | p-value |  | r | p-value |
| **Clinical parameters** |  |  |  |  |  |
| Age (y) | 0.151 | 0.145 |  | 0.057 | 0.586 |
| BMI (kg/m2) | 0.119 | 0.251 |  | 0.243 | 0.018 |
| ALT (U/l) | -0.154 | 0.136 |  | -0.039 | 0.709 |
| Fasting glucose (mmol/l) | -0.071 | 0.492 |  | -0.035 | 0.738 |
| Fasting insulin (U/l) | -0.082 | 0.448 |  | 0.083 | 0.447 |
| Total cholesterol (mmol/l) | 0.072 | 0.491 |  | 0.008 | 0.939 |
| HDL cholesterol (mmol/l) | 0.202 | 0.052 |  | -0.068 | 0.518 |
| LDL cholesterol (mmol/l) | 0.117 | 0.264 |  | 0.035 | 0.738 |
| Triglycerides (mmol/l) | -0.186 | 0.074 |  | -0.142 | 0.173 |
| Erythrocyte folate (nmol/l) | -0.331 | 0.025 |  | -0.205 | 0.173 |
| Glycine betaine | 0.118 | 0.377 |  | 0.148 | 0.268 |
| L-Methionine | -0.108 | 0.422 |  | -0.007 | 0.960 |
|  |  |  |  |  |  |
| **Liver histology** |  |  |  |  |  |
| Steatosis grade | -0.247 | 0.036 |  | -0.233 | 0.048 |
| Fibrosis stage | -0.059 | 0.624 |  | -0.037 | 0.760 |
| Lobular inflammation | -0.026 | 0.826 |  | -0.014 | 0.909 |
| Ballooning | -0.100 | 0.401 |  | -0.151 | 0.206 |
| Statistical significance calculated with Spearman correlation. Nominal p-values are presented, but none of them remained significant after correction for multiple testing using the Benjamini-Hochberg procedure with FDR 0.25. | | | | | |
|  |  |  |  |  |  |
|  |  |  |  |  |  |
|  |  |  |  |  |  |
|  |  |  |  |  |  |
