# Supplementary material for: Liver DNA methylation of FADS2 associates with FADS2 genotypex
Source: Clin Epigenetics. 2019 Jan 17;11:10. doi: 10.1186/s13148-019-0609-1 (PMC6337806; doi:10.1186/s13148-019-0609-1)
Supplement: Supplementary file 6 — Association between liver histology and DNA methylation of CpG sites annotated to FADS2 and FADS1. (DOCX 29 kb) [file 13148_2019_609_MOESM6_ESM.docx]

ADDITIONAL MATERIAL:

**Liver DNA methylation of *FADS2* associates with *FADS2* genotype.**

Paula Walle^1^, Ville Männistö^2^, Vanessa D. de Mello^1^, Maija Vaittinen^1^, Alexander Perfilyev^3^, Kati Hanhineva^1^, Charlotte Ling^3^, Jussi Pihlajamäki^1,4^

1 Department of Clinical Nutrition, Institute of Public Health and Clinical Nutrition, University of Eastern Finland, Kuopio, Finland.

2 Department of Medicine, University of Eastern Finland and Kuopio University Hospital, Finland

3 Epigenetics and Diabetes Unit, Department of Clinical Sciences, Lund University Diabetes Centre, Malmö, Sweden.

4 Clinical Nutrition and Obesity Center, Kuopio University Hospital, Finland

|  |  |  |  |  |  |
| --- | --- | --- | --- | --- | --- |
|  | **CpG site** | **Steatosis grade** | **Lobular inflammation** | **Ballooning** | **Fibrosis stage** |
|  |  |  |  |  |  |
| ***FADS2*** | **cg00603274** | 0.14 | 0.02 | -0.09 | 0.03 |
|  | **cg00614641** | 0.05 | 0.16 | 0.20 | 0.17 |
|  | **cg01400685** | 0.14 | 0.29 | -0.01 | 0.27 |
|  | **cg02563962** | 0.05 | -0.15 | -0.22 | -0.05 |
|  | **cg05698098** | 0.15 | -0.04 | -0.09 | -0.01 |
|  | **cg06781209** | -0.25 | -0.03 | -0.10 | -0.06 |
|  | **cg07005513** | -0.08 | -0.18 | -0.22 | -0.07 |
|  | **cg07591205** | 0.21 | 0.10 | -0.04 | 0.18 |
|  | **cg07999042** | -0.23 | -0.01 | -0.15 | -0.04 |
|  | **cg10868875** | -0.06 | -0.25 | -0.18 | -0.24 |
|  | **cg11250194** | -0.20 | -0.15 | -0.04 | -0.17 |
|  | **cg14911132** | 0.32 | 0.19 | 0.09 | 0.14 |
|  | **cg16576620** | 0.10 | -0.05 | -0.03 | -0.01 |
|  | **cg19610905** | 0.16 | 0.02 | 0.00 | 0.05 |
|  | **cg21709803** | -0.17 | 0.08 | -0.13 | 0.06 |
|  | **cg23760165** | -0.21 | 0.04 | 0.09 | 0.01 |
|  | **cg25303599** | -0.02 | -0.04 | -0.02 | 0.02 |
|  | **cg25324164** | -0.05 | 0.10 | -0.03 | 0.03 |
| ***FADS1*** | **cg00786201** | -0.13 | -0.14 | -0.21 | -0.13 |
|  | **cg02085160** | 0.07 | 0.00 | -0.08 | -0.07 |
|  | **cg03735013** | -0.17 | -0.24 | -0.21 | -0.23 |
|  | **cg03921599** | 0.03 | -0.03 | -0.15 | -0.04 |
|  | **cg05168842** | 0.10 | 0.02 | -0.03 | 0.11 |
|  | **cg06405978** | 0.16 | 0.01 | -0.04 | 0.01 |
|  | **cg06985934** | -0.22 | -0.21 | -0.17 | -0.26 |
|  | **cg07152460** | 0.03 | -0.10 | -0.16 | -0.11 |
|  | **cg07689907** | -0.19 | 0.03 | -0.17 | -0.02 |
|  | **cg07709195** | -0.13 | -0.02 | -0.17 | -0.04 |
|  | **cg09462826** | -0.24 | -0.08 | -0.10 | -0.15 |
|  | **cg09677638** | -0.04 | -0.17 | -0.16 | -0.07 |
|  | **cg10515671** | -0.12 | 0.07 | -0.10 | 0.03 |
|  | **cg11606466** | 0.17 | 0.14 | 0.21 | 0.18 |
|  | **cg12517394** | -0.05 | -0.20 | -0.27 | -0.11 |
|  | **cg13100764** | 0.07 | 0.23 | -0.04 | 0.25 |
|  | **cg13121120** | -0.21 | -0.11 | -0.18 | -0.14 |
|  | **cg13475388** | 0.00 | 0.00 | -0.04 | 0.05 |
|  | **cg14725641** | 0.13 | 0.02 | 0.03 | -0.01 |
|  | **cg15598662** | -0.06 | -0.01 | -0.08 | -0.03 |
|  | **cg16213375** | **-0.37** | -0.31 | -0.30 | **-0.36** |
|  | **cg16328381** | 0.05 | 0.12 | 0.05 | 0.14 |
|  | **cg23992449** | 0.00 | -0.05 | 0.07 | -0.05 |
|  | **cg24870774** | 0.00 | -0.19 | -0.17 | -0.12 |
|  | **cg25326896** | -0.09 | -0.02 | -0.14 | -0.05 |
|  | **cg25401284** | -0.22 | -0.10 | -0.12 | -0.07 |
|  | **cg25448062** | 0.00 | 0.02 | 0.31 | 0.01 |
|  | **cg25837350** | 0.11 | -0.02 | -0.11 | -0.03 |
|  | **cg27173322** | -0.04 | -0.08 | -0.19 | -0.11 |

**Additional File 6. Association between estimated delta-5 desaturase activity in serum and liver and DNA methylation of CpG sites in *FADS2* and *FADS1* (n=72).** A Spearman's correlation was run to assess the relationship between methylation levels and liver histology. Data is presented as Spearman correlation coefficient, and associations with a nominal p-value <0.05, which remained significant after correction for multiple testing using the Benjamini-Hochberg procedure with FDR 0.25, are indicated by boxes around the correlation coefficient. Positive correlations indicated with red and negative correlations with blue.
