# Supplementary material for: Liver DNA methylation of FADS2 associates with FADS2 genotypex
Source: Clin Epigenetics. 2019 Jan 17;11:10. doi: 10.1186/s13148-019-0609-1 (PMC6337806; doi:10.1186/s13148-019-0609-1)
Supplement: Supplementary file 7 — Characteristics of the groups based on erythrocyte folate. (DOCX 27 kb) [file 13148_2019_609_MOESM7_ESM.docx]

ADDITIONAL MATERIAL:

**Liver DNA methylation of *FADS2* associates with *FADS2* genotype.**

Paula Walle^1^, Ville Männistö^2^, Vanessa D. de Mello^1^, Maija Vaittinen^1^, Alexander Perfilyev^3^, Kati Hanhineva^1^, Charlotte Ling^3^, Jussi Pihlajamäki^1,4^

1 Department of Clinical Nutrition, Institute of Public Health and Clinical Nutrition, University of Eastern Finland, Kuopio, Finland.

2 Department of Medicine, University of Eastern Finland and Kuopio University Hospital, Finland

3 Epigenetics and Diabetes Unit, Department of Clinical Sciences, Lund University Diabetes Centre, Malmö, Sweden.

4 Clinical Nutrition and Obesity Center, Kuopio University Hospital, Finland

| **Additional File 7. Characteristics of the groups based on erythrocyte folate.** | | | | | | | | | | |  |  |
| --- | --- | --- | --- | --- | --- | --- | --- | --- | --- | --- | --- | --- |
|  | |  | | | | | | | | | | |
|  | **1st tertile (n=15)** | | | | **2nd tertile (n=16)** | | | | **3rd tertile (n=15)** | | | **p-value** |
|  |  | |  |  |  |  |  | |  |  |  |  |
| Age (y) | 50.5 | | ± | 8.6 | 48.9 | ± | 5.9 | | 52.3 | ± | 7.3 | 0.444 |
| BMI (kg/m^2^) | 44.3 | | ± | 6.9 | 41.7 | ± | 4.3 | | 41.8 | ± | 4.8 | 0.339 |
| ALT (U/l) | 47.5 | | ± | 38.6 | 49.7 | ± | 33.8 | | 38.3 | ± | 15.5 | 0.743 |
| Fasting glucose (mmol/l) | 6.7 | | ± | 1.6 | 6.3 | ± | 2.3 | | 5.9 | ± | 0.5 | 0.426 |
| Fasting insulin (U/l) | 21.9 | | ± | 18.4 | 15.6 | ± | 12.0 | | 18.7 | ± | 10.3 | 0.391 |
| Total cholesterol (mmol/l) | 4.4 | | ± | 1.0 | 4.1 | ± | 0.8 | | 4.3 | ± | 1.2 | 0.844 |
| HDL cholesterol (mmol/l) | 1.1 | | ± | 0.3 | 1.0 | ± | 0.2 | | 1.1 | ± | 0.3 | 0.847 |
| LDL cholesterol (mmol/l) | 2.6 | | ± | 0.7 | 2.3 | ± | 0.8 | | 2.5 | ± | 1.1 | 0.593 |
| Triglycerides (mmol/l) | 1.5 | | ± | 0.6 | 1.9 | ± | 0.7 | | 1.6 | ± | 0.7 | 0.306 |
| Erythrocyte folate (nmol/l) | 1000.7 | | ± | 203.8 | 1418.1 | ± | 91.6 | | 1983.8 | ± | 351.8 | **1x10^-13^** |
| Data presented as mean±SD. | |  |  |  |  |  |  |  | |  |  |  |
| Statistical significance calculated with ANOVA or Welch ANOVA. | | | | | | | |  | |  |  |  |
