# Supplementary material for: Liver DNA methylation of FADS2 associates with FADS2 genotypex
Source: Clin Epigenetics. 2019 Jan 17;11:10. doi: 10.1186/s13148-019-0609-1 (PMC6337806; doi:10.1186/s13148-019-0609-1)
Supplement: Supplementary file 8 — DNA methylation of FADS2 in groups based on erythrocyte folate. (DOCX 26 kb) [file 13148_2019_609_MOESM8_ESM.docx]

ADDITIONAL MATERIAL:

**Liver DNA methylation of *FADS2* associates with *FADS2* genotype.**

Paula Walle^1^, Ville Männistö^2^, Vanessa D. de Mello^1^, Maija Vaittinen^1^, Alexander Perfilyev^3^, Kati Hanhineva^1^, Charlotte Ling^3^, Jussi Pihlajamäki^1,4^

1 Department of Clinical Nutrition, Institute of Public Health and Clinical Nutrition, University of Eastern Finland, Kuopio, Finland.

2 Department of Medicine, University of Eastern Finland and Kuopio University Hospital, Finland

3 Epigenetics and Diabetes Unit, Department of Clinical Sciences, Lund University Diabetes Centre, Malmö, Sweden.

4 Clinical Nutrition and Obesity Center, Kuopio University Hospital, Finland

| **Additional File 8. DNA methylation of *FADS2* in groups based on erythrocyte folate.** | | | | | | | | | | | | | | | |
| --- | --- | --- | --- | --- | --- | --- | --- | --- | --- | --- | --- | --- | --- | --- | --- |
|  | |  | | | | | | | | | | |  |  | |
|  | **1st tertile (n=15)** | | | | **2nd tertile (n=16)** | | | | **3rd tertile (n=15)** | | | | | | **ANOVA* p-value** |
| **cg00603274** | 0.05 | | ± | 0.01 | | 0.05 | ± | 0.01 | 0.05 | ± | 0.01 |  | 0.266 | | |
| **cg00614641** | 0.06 | | ± | 0.01 | | 0.05 | ± | 0.01 | 0.05 | ± | 0.01 |  | 0.576 | | |
| **cg01400685** | 0.36 | | ± | 0.06 | | 0.33 | ± | 0.06 | 0.29 | ± | 0.09 | ^a^ | **0.045** | | |
| **cg02563962** | 0.05 | | ± | 0.01 | | 0.06 | ± | 0.01 | 0.05 | ± | 0.01 |  | 0.065 | | |
| **cg05698098** | 0.10 | | ± | 0.02 | | 0.11 | ± | 0.02 | 0.11 | ± | 0.01 |  | 0.247 | | |
| **cg06781209** | 0.11 | | ± | 0.04 | | 0.09 | ± | 0.04 | 0.08 | ± | 0.05 |  | 0.050 | | |
| **cg07005513** | 0.05 | | ± | 0.01 | | 0.05 | ± | 0.01 | 0.05 | ± | 0.01 |  | 0.236 | | |
| **cg07591205** | 0.92 | | ± | 0.01 | | 0.93 | ± | 0.01 | 0.93 | ± | 0.01 |  | 0.592 | | |
| **cg07999042** | 0.90 | | ± | 0.04 | | 0.89 | ± | 0.05 | 0.87 | ± | 0.05 |  | 0.085 | | |
| **cg10868875** | 0.04 | | ± | 0.01 | | 0.04 | ± | 0.01 | 0.04 | ± | 0.00 |  | 0.401 | | |
| **cg11250194** | 0.87 | | ± | 0.03 | | 0.86 | ± | 0.03 | 0.88 | ± | 0.02 |  | 0.159 | | |
| **cg14911132** | 0.09 | | ± | 0.01 | | 0.09 | ± | 0.02 | 0.10 | ± | 0.02 |  | 0.225 | | |
| **cg16576620** | 0.03 | | ± | 0.01 | | 0.03 | ± | 0.00 | 0.03 | ± | 0.00 |  | 0.808 | | |
| **cg19610905** | 0.05 | | ± | 0.01 | | 0.05 | ± | 0.01 | 0.05 | ± | 0.01 |  | 0.740 | | |
| **cg21709803** | 0.10 | | ± | 0.03 | | 0.08 | ± | 0.03 | 0.08 | ± | 0.03 |  | 0.111 | | |
| **cg23760165** | 0.03 | | ± | 0.01 | | 0.03 | ± | 0.01 | 0.03 | ± | 0.01 |  | 0.611 | | |
| **cg25303599** | 0.05 | | ± | 0.01 | | 0.05 | ± | 0.01 | 0.05 | ± | 0.01 |  | 0.181 | | |
| **cg25324164** | 0.27 | | ± | 0.04 | | 0.23 | ± | 0.05 | 0.25 | ± | 0.06 |  | 0.135 | | |
| Data presented as mean±SD | | | | | | | | | | | | | | | |
| *one-way ANOVA or Welch ANOVA | | | | | | | | | | | | | | | |
| *^a^* p<0.05 compared to 1st tertile in Bonferroni post hoc analysis | | | | | | | | | | | | | | | |
