# Supplementary material for: Liver DNA methylation of FADS2 associates with FADS2 genotypex
Source: Clin Epigenetics. 2019 Jan 17;11:10. doi: 10.1186/s13148-019-0609-1 (PMC6337806; doi:10.1186/s13148-019-0609-1)
Supplement: Supplementary file 9 — Association between cg06781209, cg07999042 and rs174616 in the FADS gene cluster. (DOCX 135 kb) [file 13148_2019_609_MOESM9_ESM.docx]

ADDITIONAL MATERIAL:

**Liver DNA methylation of *FADS2* associates with *FADS2* genotype.**

Paula Walle^1^, Ville Männistö^2^, Vanessa D. de Mello^1^, Maija Vaittinen^1^, Alexander Perfilyev^3^, Kati Hanhineva^1^, Charlotte Ling^3^, Jussi Pihlajamäki^1,4^

1 Department of Clinical Nutrition, Institute of Public Health and Clinical Nutrition, University of Eastern Finland, Kuopio, Finland.

2 Department of Medicine, University of Eastern Finland and Kuopio University Hospital, Finland

3 Epigenetics and Diabetes Unit, Department of Clinical Sciences, Lund University Diabetes Centre, Malmö, Sweden.

4 Clinical Nutrition and Obesity Center, Kuopio University Hospital, Finland


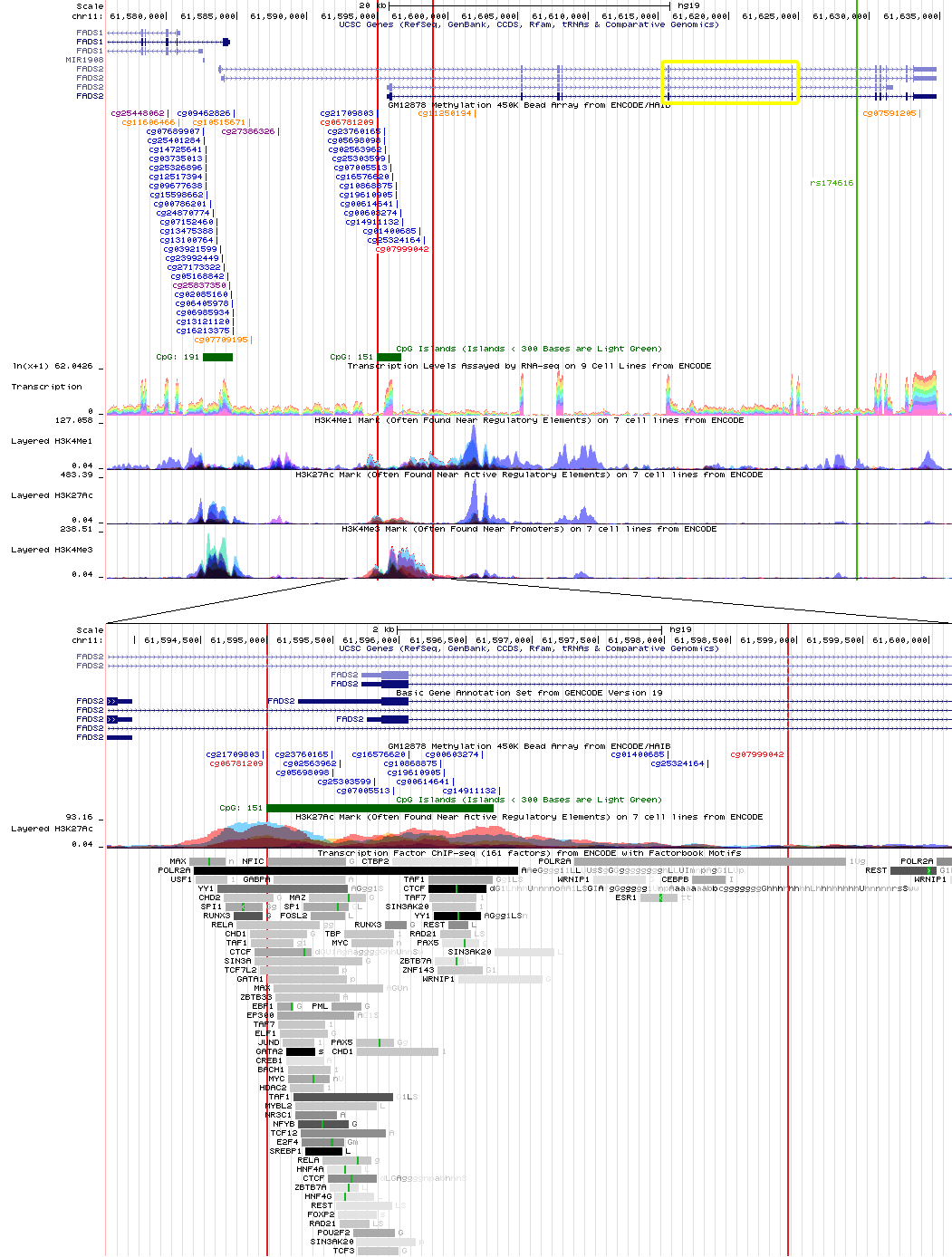
**Additional file 9. Association between cg06781209, cg07999042 and rs174616 in the *FADS* gene cluster.** CpG sites selected for analysis are marked with red lines. The *FADS2* gene variant rs174616 is marked with green line. The lower panel shows more closely the association between selected CpG sites and transcription factor binding sites. cg06781209 in located in a CpG island within the first 1500 bp upstream from the transcription start site, in a potential gene enhancer region indicated by monomethylation of histone H3 lysine4 (H3K4me1) and acetylation of H3K27 (H3K27Ac). cg06781209 is in an area with several transcription factor binding sites: POLR2A, YY1, CHD2, RELA, CHD1, TAF1, CTCF, SIN3A, and TCF7L2. High peaks of H3K27Ac indicate activation of transcription in this area. cg07999042 is located in the body of *FADS2*, and it is in the same area as transcription factor binding site for POLR2A. Figure modified from the ENCODE data in the UCSC Genome Browser.
